# Expression of Concern: MiR-942 regulates the function of breast cancer cell by targeting FOXA2

**DOI:** 10.1042/BSR-20192298_EOC

**Published:** 2021-04-07

**Keywords:** Breast cancer, FOXA2, MicroRNA-942, Prognosis, Progression

The Editorial Office has been made aware of potential issues surrounding the scientific validity of this paper, hence has issued an expression of concern to notify readers whilst the Editorial Office investigates.

